# Efficacy of Cognitive Behavioral Therapy for Anxiety-Related Disorders: A Meta-Analysis of Recent Literature

**DOI:** 10.1007/s11920-022-01402-8

**Published:** 2022-12-19

**Authors:** Shalini Bhattacharya, Carmen Goicoechea, Saeideh Heshmati, Joseph K. Carpenter, Stefan G. Hofmann

**Affiliations:** 1 Department of Clinical Psychology and Psychotherapy, Philipps-University of Marburg, Marburg, Germany; 2 Department of Experimental Psychology, University of Granada, Granada, Spain; 3 Department of Psychology, Claremont Graduate University, Claremont, CA USA; 4 National Center for PTSD Women’s Health Sciences Division, VA Boston Healthcare System, Boston, MA USA; 5 Department of Psychiatry, Boston University School of Medicine, Boston, MA USA; 6 Department of Psychological and Brain Sciences, Boston University, Boston, MA USA

**Keywords:** Anxiety, Anxiety disorders, Cognitive behavioral therapy, Meta-analysis, Posttraumatic stress disorder, Randomized controlled trials

## Abstract

**Purpose of Review:**

Effective treatment of anxiety-related disorders is crucial, considering the prevalence of such disorders and their association with poor psychosocial functioning. To evaluate the most recent evidence on the efficacy of cognitive behavioral therapy (CBT) for anxiety-related disorders in adults, we conducted a meta-analysis of randomized placebo-controlled trials published since 2017.

**Recent Findings:**

Ten studies with a total of 1250 participants met the inclusion criteria. Seven of these studies examined PTSD. The findings demonstrated small placebo-controlled effects of CBT on target disorder symptoms (Hedges’ *g* = 0.24, *p* < 0.05) and depression (Hedges’ *g* = 0.15, *p* = n.s). When examining only PTSD studies, effects were reduced (Hedges’ *g* = 0.14,* p* < 0.05). Heterogeneity in most analyses was very low, and no publication bias was found.

**Summary:**

Effect sizes from placebo-controlled trials from the past 5 years appear to be smaller than those in prior meta-analyses. The findings are largely driven by research on PTSD, with few placebo-controlled trials of other anxiety-related disorders published since 2017.

**Supplementary Information:**

The online version contains supplementary material available at 10.1007/s11920-022-01402-8.

## Introduction

Anxiety disorders are highly disabling and have a significant impact on patient's quality of life, relationships, occupational, and social abilities [1–5]. In addition, such disorders contribute to enormous economic and public health costs [6, 7]. A recent report from the Global Burden of Disease Study estimates that more than 301 million people globally are affected by anxiety [8]. A global return on investment analysis report indicates that approximately 12 billion workdays per year are lost due to anxiety and depressive disorders [9]. Among the classifications of anxiety disorders, specific phobia (10.3%), panic disorders (6%), social phobia (2.7%), and generalized anxiety disorders (GAD) (2.2%) are the most common [10]. In the current classification of anxiety disorders under the Diagnostic and Statistical Manual of Mental Disorders (DSM-5), obsessive compulsive disorder (OCD), acute stress disorder (ASD), and posttraumatic disorder (PTSD) are no longer classified as anxiety disorders; however, they are highly comorbid with similar characteristics to anxiety symptoms such as irrational fear, avoidance and hyperarousal [11–14]. Clinical guidelines recommend psychological and pharmacotherapy as a first-line treatment for anxiety-related disorders [15–21]. Despite evidence for the efficacy of such interventions, a substantial proportion of patients receiving treatment still remain symptomatic [22].

The most extensively researched and tested psychotherapy is cognitive–behavioral therapy (CBT) [23]. CBT is considered the gold standard evidence-based intervention for treating anxiety disorders [24••, 25, 26]. The main aim of CBT-based intervention is to alter maladaptive emotional responses by challenging dysfunctional thinking patterns [27]. Several meta-analytic reviews of CBT have found large effects and concluded that CBT effectively treats anxiety disorders [23, 28–30]. However, the magnitude of these effects is influenced by the studies’ comparison conditions, such as waitlist (WL) or treatment as usual (TAU) [31, 32]. A limitation of TAU as a control condition is that it tends to be heterogeneous and not structurally equivalent, both within and between studies [33]. A WL control, on the other hand, does not control for nonspecific factors such as patient expectations of the treatment outcomes or therapeutic alliance [34]. Moreover, study samples may be biased by only selecting patients who are willing to be randomized to a waitlist [35, 36]. As a result, TAU and WL comparators are suboptimal, potentially inflating estimates of treatment efficacy [37, 38]. A more systematic approach to address this problem is to compare an active intervention with a psychological or pill placebo [26, 39, 40]. Such comparisons allow for an examination of the specific effects of the intervention beyond factors common across treatments [41]. Pill placebos serve the purpose of controlling for patient expectations of improvement, and control for some level of interaction with a clinician [42].

In general, psychological placebos are intended to mimic the structure of the active interventions, controlling for nonspecific factors including the frequency of therapist interaction, without including active treatment ingredients such as cognitive restructuring and behavioral interventions [43]. Recent evidence on nondirective supportive therapy [NDST] shows positive effects compared to WL and TAU but is less effective compared to psychological treatments [44]. NDST treatment follows unstructured therapy, with the main aim of offering support through active listening [45, 46]. Another form of a psychological placebo is present-centred therapy (PCT), which controls for nonspecific factors in psychotherapy [47]. The main component of PCT includes psychoeducation and strategies to address stressors in a nondirective manner [48]. In recent years, PCT has been effective in reducing PTSD severity as compared to WL [49••].

Two meta-analytic reviews have been published that specifically examined placebo-controlled trials of CBT for adults with anxiety-related disorders. In 2008, Hofmann and Smits [26] compiled data from 27 studies examining anxiety disorders, obsessive compulsive disorder, and PTSD, reporting a large effect size (Hedges’ *g* = 0.73) of CBT compared to placebo. In 2018, Carpenter et al. [29] updated this meta-analysis with an additional 16 studies, finding a moderate placebo-controlled effect size (Hedges’ *g* = 0.56). Together, these studies highlighted the strong support for CBT as an efficacious intervention for anxiety-related disorders, even when using more rigorous comparison conditions, though they also found more modest effect sizes than meta-analysis comparing CBT against WL controls [50]. The present study aimed to update the prior two meta-analyses on placebo-controlled trials of CBT for adults with anxiety-related disorders conducted by Hofmann and Smits [26] and Carpenter et al. [29] Such an update can provide additional insight into the size of intervention effects specific to CBT for adults based on the most recent literature, further informing treatment recommendations for those living with anxiety. To be consistent with the prior analysis, we included OCD, ASD, and PTSD, although they are no longer classified as anxiety disorders.

## Method

### Design

This paper was designed as a meta-analysis focusing on recent randomized controlled clinical trials (RCTs) comparing primary outcomes of CBT for anxiety-related disorders in adults with placebo control conditions (psychological or pill). Studies were selected by the first and the second author (SB, CG), and disagreements were resolved through discussion with a third researcher (SH). We followed the same eligibility criteria as Hofmann and Smits [26] and Carpenter et al. [29]. This study protocol was prospectively registered in the Open Science Framework (https://osf.io/t9whj).

### Search Strategy

We searched three major bibliographical databases (PubMed, PsycINFO, Web of Science) to identify studies published from January 1, 2017, to January 31, 2022. We used the following search terms indicative of studies with CBT conditions: (((random*)) AND (((cognitive behavior* therap*) OR (cognitive therap*) OR (behavior* therap*))) AND ((GAD) OR (generalized anxiety disorder) OR (OCD) OR (obsessive compulsive disorder) OR (social phobia) OR (social anxiety disorder) OR (specific phobia) OR (simple phobia) OR (PTSD) OR (posttraumatic stress disorder) OR (panic disorder) OR (acute stress disorder))) NOT (children).

### Inclusion and Exclusion Criteria

Studies were included in the present meta-analysis if (1) patients were between ages 18 and 65 and met DSM-III-R, DSM-IV, or DSM-5 diagnostic criteria for acute stress disorder, GAD, OCD, PTSD, SAD, or specific phobia as determined by a psychometrically sound and structured diagnostic instrument; (2) patients had to be randomly assigned to either CBT or placebo (pill or psychological). Psychological placebos are defined as nondirective and nonspecific psychological interventions, including discussion and interaction of patient’s problems with the therapist [41]; (3) the severity of anxiety symptoms was assessed through a validated clinical interview or self-report instrument administered pre- and posttreatment; (4) studies provided sufficient data to calculate effect sizes.

Studies were excluded if (1) patients had medical comorbidities (e.g., substance abuse or a medical condition); (2) the intervention was delivered in part or fully by a computerized or internet-based program rather than by a therapist, (see [51, 52] for recent meta-analyses on internet-based CBT for anxiety) (3) studies consisted of secondary analyses of previously published datasets; (4) active placebo intervention targeted problems such as self-guided exposures; or (5) the intervention included “third-wave” (acceptance and commitment therapy, mindfulness-based interventions, etc.), given that interventions involved in such treatments such as mindfulness and acceptance exercises go beyond the core strategies of CBT, which focus on cognitive restructuring and exposure [53]. No language restrictions were applied.

### Data Extraction

Two researchers independently conducted data extraction for the meta-analysis (SB, CG). The following data were extracted: (1) characteristics of the studies: sample size, type of placebo condition, year of publication, type of analysis (completer or intention to treat (ITT)); (2) characteristics of the intervention: type of CBT (exposure, cognitive or both), format (group and individual, number of sessions); (3) characteristics of the participants (demographics); and (4) postintervention and follow-up outcome data on anxiety symptoms, depression, and quality of life. When a study reported more than one instrument to measure target disorders, we averaged the effect size from the instruments to obtain a more accurate result.

### Data Synthesis

All analyses were conducted in R using the “metafor” package (version 3.6.2) [54, 55]. We first calculated the effect size (Hedges’ *g*) indicating the difference between the CBT and placebo groups at posttreatment. Separate meta-analyses were conducted for disorder specific symptoms and other anxiety symptoms such as PTSD and depression. To calculate the effect size for the outcomes, we used the mean, standard deviation, and the number of participants from the CBT and placebo groups [56, 57]. Effect sizes were calculated as the difference in means between the treatment and control groups divided by the pooled standard deviation [58]. If the studies did not report the mean or standard deviation, we used other statistics, such as change scores, binary outcomes, and *t* test statistics to calculate the effect size. The pooled effect size was calculated by combining effect sizes from the individual studies through random effects models using the Hartung-Knapp-Sidik-Jonkman (HKSJ) adjustment [59, 60]. The indicative effect size for the interpretation of Hedges’ *g* is small effect 0.20, medium effect 0.50, and large effect 0.80 [61].

For meta-analyses based on dichotomous outcomes such as dropout rates, we calculated odds ratios (ORs) and their 95% confidence intervals using the Cox–Hinkley–Miettinen–Nurminen method [62]. An OR of 1 indicates that the event is unlikely to occur in either group. An OR greater than 1 indicates a greater likelihood of dropout in CBT compared to placebo. In our analysis, dropout was defined as the number of participants who started the treatment but did not complete the full treatment protocol [63].

To examine the homogeneity of the effect sizes, we calculated the *I*^2^ statistic and its 95% confidence interval (CI). A value of 0% indicates that the effects are homogenous, 25% indicates low heterogeneity, 50% moderate heterogeneity, and a value over 75% suggests high heterogeneity [64].

We conducted subgroup analyses using mixed effect models [65]. In this method, the studies within the subgroups are pooled with a random effect model, while the test for difference between the groups is conducted with a fixed effects model. We performed five subgroup analyses: (1) treatment format (individual vs. group therapy), (2) analysis type (completer vs. ITT), (3) mode of assessment (self-report vs. clinician report), (4) characteristics of the participants (veteran or active-duty military participants vs. non-military participants), and (5) comparison condition (PCT vs. other psychological placebo). We conducted this latter comparison given accumulating evidence that despite being designed as a placebo control, PCT may be an effective stand-alone treatment, thereby deflating effect sizes of CBT when used as a control [49••].

Sensitivity analyses were conducted by excluding potential outliers and recalculating the effect size. Outliers were defined as studies for which the 95% CI of the effect sizes did not overlap with the 95% CI of the pooled effect sizes [66].

Meta regression analyses were used to investigate the impact of the number of therapy sessions on treatment outcomes. The regression coefficient indicates the strength of the relationship and along with the *p* value; they can inform whether there was a linear relationship between the two variables [67].

Publication bias was examined by inspecting the funnel plot and Duval and Tweedie’s trim and fill procedure [68]. The presence of publication bias was tested through Egger’s test for the asymmetry of the funnel plot [69].

### Risk of Bias Assessment

The study quality was assessed by two independent researchers using the Cochrane risk of bias assessment tool version 1 (RoB) [70]. The tool involves four criteria: (1) adequate generation of randomization sequence; (2) allocation concealment; (3) blinding of assessors; (4) appropriate methods for handling missing data (rated as positive for intention-to-treat analyses), indicating that all patients at baseline randomizations were included in the analyses; and (5) selective outcome reporting. An individual item was rated as 0 indicating studies with low risk, 1 indicating those with unclear bias risk, and 2 indicating those with high risk. If a study had insufficient information regarding the items, they were classified as having an unclear risk of bias. To determine the overall quality of the study, each individual item score was added, and a study with a score of less than 2 was classified as low risk, while a study with a score more than 2 was considered high risk.

## Results

### Study Sample Characteristics

**Fig. 1:**
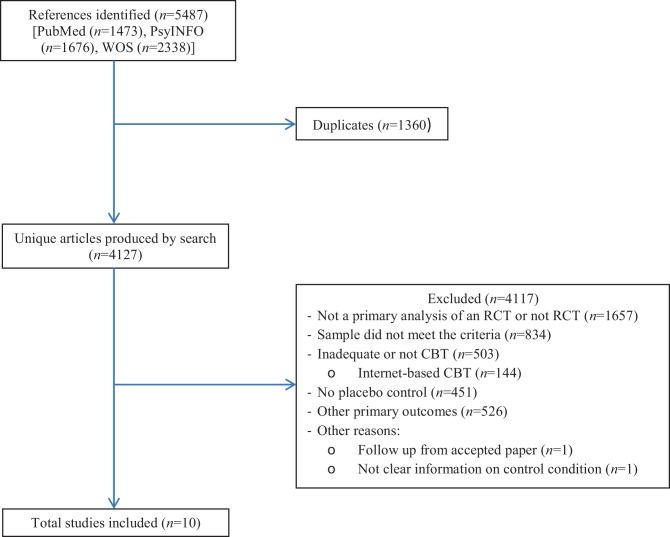
Flow diagram of study selection process

Figure 1 presents a flow diagram illustrating the study selection process. The final analysis included 10 studies, and a total of 1250 patients were randomized to the CBT (701 patients) or placebo (549 patients) condition. The characteristics of the 10 included studies are presented in Table 1. The average study sample had a mean age of 39.91 years (SD = 9.49), and 41.95% were female participants (SD = 32.81). Of the studies that reported race, 60% of patients were White or Caucasian (*n* = 7 studies) (SD = 9.87), 23% were Black participants (*n* = 7 studies) (SD = 13.75), and 3.95% were Asian participants (*n* = 6 studies) (SD = 3.19). The majority of studies examined the treatment of PTSD (*n* = 7 studies), while we found one study on ASD, GAD, and SAD, and no studies of panic disorder, OCD, or specific phobia. Of the CBT treatments, 3 studies used exposure techniques, 2 studies focused on cognitive strategies, and 5 included both elements in their interventions. The format of treatment delivery was 4 studies involving individual therapy and 6 conducting group therapy. The mean duration of treatments was 11.4 sessions (SD = 3.69). Seven studies used measures of depression at posttreatment, and three studies reported measures of quality of life. Regarding follow-up measures, 7 of the 10 studies reported treatment effects 6 months after posttreatment. No studies were found that used pill placebo as a control condition. Of the psychological placebo conditions, the most frequent was present-centred therapy (*n* = 4 studies), followed by psychoeducation (*n* = 3 studies) and other psychological placebos (*n* = 3 studies).

**Table 1:** Descriptions and characteristics of studies included in the meta-analysis

**Study**	**Disorder**	**CBT**	**PBO**	**Other comparison conditions**	**Total N (CBT + PBO)**	**# Sessions**	**Symptom measures**	**Other anxiety measures**	**Dep. measures**	**QOL measures**	**Analysis type**	**Mean age**	**% Fem**	**% White**	**% Black**	**% Asian**
Fauerbach et al. 2020 [71•]	ASD	Ind C + E	Nondirective supportive psychotherapy	n/a	28	4	DTS	n/a	PHQ-9	n/a	Completer	39	44.6	52.5	40	2.5
Simon et al. 2021 [72•]	GAD	Grp C + E	Stress education	Kundalini yoga	133	12	CGI-I	n/a	n/a	n/a	ITT	33.35	68.4	74.43	6.01	9.77
Carlsoon et al. 2018 [73•]	PTSD	Grp C + E	Stress management	n/a	126	16	HTQ	HAM-A	HAM-D	WHO-5, GAF, SDS	ITT	43.3	43.6	n/a	n/a	n/a
Foa, et al. 2018* [74•]	PTSD	Ind E only	Present-centered therapy	Minimal-contact control	326	10	PCL-S, PSS-I	n/a	n/a	n/a	ITT	32.69	12.9	60.42	24.23	0.92
Haynes et al. 2020* [75••]	PTSD	Grp C only	Present-centered therapy	n/a	37	12	CAPS, PCL-M	n/a	HAM-D	n/a	Completer	48.42	0	55.81	6.97	2.32
Johnson et al. 2020 [76••]	PTSD	Grp C only	Present-centered therapy	n/a	142	16	CAPS	n/a	CES-D	SF-12	ITT	35.1	100	46.51	44.18	n/a
Nidich et al. 2018* [77•]	PTSD	Ind E only	Psychoeducation	Transcendental meditation	134	12	CAPS, PCL-M	n/a	PHQ-9	n/a	ITT	47.35	16.4	55.97^1^	23.88	6.71
Sloan et al. 2018* [78•]	PTSD	Grp C + E	Present-centered therapy	n/a	198	14	CAPS-5, PCL-5	BAI	BDI-II	SF-36	ITT	55.82	0	74.2	16.7	1.5
Vera et al. 2022 [79••]	PTSD	Ind E only	Applied relaxation	n/a	76	12	CAPS-5, PCL-5	STAI-S	PHQ-9	n/a	Completer	43.62	81.6	n/a	n/a	n/a
Samantaray et al. 2021 [80••]	SAD	Grp C + E	Psychoeducational—supportive therapy	n/a	50	6	LSAS, SPIN	n/a	n/a	n/a	ITT	20.52	52	n/a	n/a	n/a
		Ind = 4 Grp = 6 C + E = 5 C only = 2 E only = 3				*M* = 11.4 SD = 3.69					Completer only = 3 ITT = 7	*M* = 39.91 SD = 9.49	*M* = 41.95% SD = 32.81	M = 59.97% SD = 9.87	M = 23.13% SD = 13.75	M = 3.95% SD = 3.19

*ASD* Acute stress disorder, *GAD* generalized anxiety disorder, *PTSD* posttraumatic stress disorder, *SAD* social anxiety disorder, *CBT* cognitive behavioral therapy, *Ind* individual, *Grp* group, *C* cognitive techniques, *E* exposure techniques, *PBO* placebo, *MCC* minimum control condition, *BAI* Beck anxiety inventory, *BDI-II* Beck depression inventory—II, *CAPS* clinician administered PTSD scale, *CAPS-5* clinician administered PTSD scale for DSM-5, *CES-D* center for epidemiologic studies depression scale, *CGI-I* clinical global impression of improvement, *DTS* Davidson trauma scale, *GAF* global assessment of function, *HAM-A* Hamilton anxiety rating scale, *HAM–D* Hamilton depression rating scale, *HTQ* Harvard trauma questionnaire, *LSAS* Liebowitz social anxiety scale, *PCL-5* PTSD symptom checklist for DSM-5, *PCL-M* PTSD checklist-military version, *PCL-S* PTSD checklist–stressor-specific, *PHQ-9* patient health questionnaire-9, *PSS-I* post-traumatic symptom scale interview, *QOL* quality of life scale; *SDS* Sheehan disability scale, *SF-12* short form health survey, *SF-36 SF* short form 36 social functioning component, *SPIN* social phobia inventory, *STAI-S* state–trait anxiety inventory–state, *WHO-5* World Health Organization Well-Being Index – 5, *ITT* intention-to-treat analyses, *Fem* female

*Study whose participants are military personnel

### Effects of CBT on Anxiety-Related Disorders

**Fig. 2:**
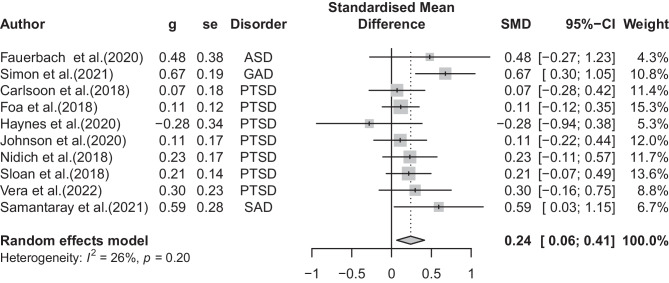
Studies examining the effects of CBT to placebo for anxiety-related disorders

The overall effect of CBT compared to placebo control across all studies at posttreatment was small, but significant *(*Hedges’ *g* = 0.24, 95% CI 0.06 to 0.41). Heterogeneity was low and significant (*I*^2^ = 26%, 95% CI 0.0 to 64%, *p* < 0.05). There was no indication of outliers. The results of these studies are summarized in Fig. 2.

Subgroup analyses were then conducted to explain this variance across studies. We found significant group differences between studies comparing CBT to PCT (Hedges’ *g* = 0.11, 95% CI − 0.11 to 0.34, *p* < 0.05) and those comparing against other psychological placebos (Hedges’ *g* = 0.36, 95% CI 0.09 to 0.62). We found no significant group differences between the following: group versus individual therapy, self-report versus clinician report, and completers versus ITT.

We found that the effects of CBT for anxiety-related disorders were very small and not significant at the 6-month follow-up (Hedges’ *g* = 0.09, 95% CI − 0.08 to 0.28, *p* = n.s.). We also ran the post-treatment analysis including 7 studies that had reported follow-up data to observe if improvements declined at follow-up. The effects for those 7 studies were slightly higher compared to 6-month follow up, albeit not significant (Hedges’ *g* = 0.20, 95% CI − 0.04 to 0.45, *p* = n.s.).

The effects of these interventions on depression were very small and non-significant (Hedges’ *g* = 0.15, 95% CI − 0.11 to 0.40). Heterogeneity was low (*I*^2^ = 36%) and not significant. We did not have enough studies to conduct any analyses for quality of life (*n* = 3). (Data presented in Table 2 of supplementary material).

### Effects of CBT on PTSD

When restricting the meta-analysis to the seven studies examining PTSD treatment, the posttreatment effect size was significant but small, Hedges’ *g* = 0.14 (95% CI 0.02 to 0.24). Heterogeneity was low and significant (*I*^2^ = 0%, 95% CI 0.0 to 71%, *p* < 0.05). The effect of CBT for PTSD studies on depression was not significant, Hedges’ *g* = 0.09 (95% CI − 0.12 to 0.32, *p* = n.s.). For PTSD studies, we found no significant group differences between PCT and other psychological placebos and for military and non-military participants (see Table 2 in supplementary materials).

### Dropout Rates

Examination of dropout rates showed a significantly greater dropout in CBT (*n* = 159 patients) compared to placebo (*n* = 98 patients (OR = 1.35, 95% CI = 1.01 to 1.78, *p* < 0.05)). The weighted mean dropout rate across all studies for CBT was 22% and 17% for placebo. In the PTSD studies (*n* = 7), the difference in dropout between CBT (21%) and placebo (15%) was significant (OR = 1.50, 95% CI = 1.08 to 2.06, *p* < 0.05).

### Metaregression

In a meta-regression analysis with the effect size on anxiety-related disorders as the dependent variable and the number of sessions as the predictor, we found no significant association between the two (*p* = n.s.).

### Publication Bias

In Duval and Tweedie’s trim and fill procedure, the adjusted effect size was identical to the main analyses, with no studies missing. Egger’s test did not indicate the presence of funnel plot asymmetry and was not significant (*p* = n.s.). This result indicates that publication bias did not have a significant effect on the summary effect size.

### Risk of Bias

Overall, the risk of bias present in the design was relatively low. The number of studies with a low, unclear and high risk of bias in each of the categories was as follows: sequence generation (10 low, 0 unclear, 0 high); allocation concealment (8 low, 1 unclear, 1 high); blinding (8 low, 0 unclear, 2 high); incomplete outcome data (7 low, 0 unclear, 3 high); selective outcome reporting (9 low, 1 unclear, 0 high). Three studies met all five predefined quality criteria, and another six met four of the five criteria [70].

## Discussion

This systematic review is the continuation of two previous works of Hofmann and Smits [26] and Carpenter et al. [29], with which it shares the objective of summarizing the state of the evidence of CBT for anxiety and related disorders based on randomized placebo-controlled trials. Our search found 10 placebo-controlled randomized trials published since 2017 and not included in Carpenter et al. [29], seven of which examined PTSD. Accordingly, this updated analysis is most informative with regard to the effects of CBT for PTSD found in the recent literature rather than anxiety-related disorders as a whole. The pooled placebo-controlled effect size for PTSD studies was statistically significant but small (Hedges’ *g* = 0.14), and notably smaller than the results reported in Carpenter et al. [29] (Hedges’ *g* = 0.41). This pattern of results does not support the notion that CBT is substantially more effective at reducing PTSD symptoms than therapy modalities designed to account for nonspecific factors of psychotherapy, at least when examining literature published since 2017. When including all anxiety-related studies in the analysis, the pooled results presented here similarly reflected a somewhat smaller effect (Hedge*s*’* g* = 0.24) compared to prior literature (Hedges’ *g* = 0.56) [29], with the non-PTSD studies demonstrating similar effect sizes individually as the prior meta-analyses [71•, 72•, 80••] (Hedges’ *g* = 0.48–0.67). We did not find any significant advantage of CBT over placebo on depression symptoms, either amongst PTSD studies or across all anxiety-related disorders, supporting the specificity of the interventions.

The minimal advantage of CBT over psychological placebo treatments for PTSD is somewhat surprising given that trauma-focused CBTs are recommended as front-line treatments in numerous treatment guidelines [18, 81]. Potential contributors to the reduced between-group effect sizes relative to prior research include (1) more rigorous research designs, as evidenced by low risk of bias ratings across the studies analyzed; (2) inclusion of two studies that did not employ trauma-focused CBT [75••, 76••] (i.e., did not involve processing of traumatic memories); (3) a substantial portion of studies examining military or veteran samples (57%) and/or group CBT (57%), both of which are associated with smaller treatment effects [82, 83], and (4) more active or effective control conditions (e.g., PCT, applied relaxation). However, the present results are in line with a meta-analysis by Belsher et al. [49••], which found that PCT, considered a placebo control in the current analysis, is non-inferior compared to trauma-focused CBT. Thus, there is a clear need for continued research on how to improve the efficacy of CBT for PTSD.

Another important finding was related to patient dropout. Participants receiving CBT for anxiety and PTSD showed a significantly higher chance of dropping out from the study than those receiving the psychological placebo. This finding is consistent with that of Carpenter et al. [29], who found higher dropout rates in the CBT condition than in the placebo condition (OR = 1.82, *p* < 0.01). A potential explanation could be that patients receiving the intervention, such as exposure-based treatment, are required to revisit the traumatic memory, which could confer the risk of early dropout [84–86]. We observed that the dropout rate for PTSD across clinical trials had a high degree of variability, which could be due to sampling error or characteristics of the study or due to distinct characteristics of the patient population, including other comorbidities [63, 86].

## Limitations

The most significant limitation to this meta-analysis is the small number of studies examining CBT for disorders other than PTSD, which precludes our ability to make conclusions about effects on anxiety-related disorders as a whole or to compare the efficacy of CBT for the various anxiety disorders, including PD, GAD, OCD, and SAD outcomes. Moreover, although heterogeneity and the risk of bias were low, the meta-analysis as a whole was based on a relatively small number of studies.

## Future Directions

Previous research suggested that skills acquired during the treatment do not improve beyond 12 months of follow-up [24••]. In our meta-analysis, only 2 studies reported a follow up of 12 months. Therefore, to substantiate the treatment effect, future trials should report a follow-up of more than 12 months for both anxiety and depression. Second, it is important that CBT continue to be tested in more diverse populations to maximize the generalizability of results. Although the studies in this analysis that reported race data demonstrated some degree of racial diversity (*M* = 40% non-White), certain racial groups (e.g., Asian participants) were not well-represented, and most [*n* = 9 studies] were set in the USA or Europe. Therefore, future trials should include more diverse samples to understand the effectiveness of the intervention in a more inclusive way. Third, regarding the control condition in RCTs, there is increasing evidence that TAU are suboptimal control conditions, as they are associated with a type 1 error resulting in an overestimation of effect size [32, 35]. Although we identified an additional 10 placebo controlled RCTs published since Carpenter et al. [29] (a 25% increase) more work is needed to establish the feasibility of including psychological placebo in RCTs as one of the most robust tests of treatment effectiveness.

## Conclusion

Randomized controlled trials published in the last 5 years show relatively minimal advantage in CBT over psychological placebos in the treatment of PTSD, though this effect may depend on the specific comparison condition. The stagnation (and even deterioration) of effect sizes over the years is an issue that is worth exploring further. Although CBT is significantly better than placebo, more efficacious treatments are needed. Anxiety disorders are highly comorbid warranting a transdiagnostic approach to identify underlying psychological processes rather than targeting the symptom constellations. Additionally, a latent disease model that drives interventions such as CBT relies on methodological factors that create limitations in finding the best treatments:(1) focusing on groups (as opposed to individuals) as the level of analysis, and (2) overlooking dynamic changes. For example, the latent disease model used in the studies examined in the current meta-analysis neglects the ergodic error [87] by clustering people based on symptoms identified at the level of the collective. This approach also overlooks the potential non-linear trajectory of change. Using analytical assessments that are limited in identifying feedback loops and non-linear changes in processes restricts our understanding of the structure of a system and how it behaves as a result of clinical inputs. When perturbations are caused in a system as a result of a clinical intervention, the system may not change linearly; in fact, change can happen in dynamic ways with complex processes that include outputs circling back into the system as inputs turning into feedback loops that we may fail to examine. A process-based approach [88••], on the other hand, takes an idiographic stance towards treatments such that each individual is examined based on contextualized and dynamic psychological processes of change. This approach allows for identifying individual moderators that work best for the given client instead of relying on treatments assigned to problem types. Taking a process-based approach may in fact be a solution to the limitations and the low effect sizes of CBT found in the current meta-analysis. We believe that the process-based approach may assist the clinician in detecting the key processes relevant to the specific needs of the client and respectively implementing the appropriate interventions targeted at those needs. Future research is warranted in further examining such a process-based approach for anxiety-related disorders.

## Supplementary Information

Below is the link to the electronic supplementary material.

Supplementary file1 (DOCX 23 KB)

## Data Availability

The data that support the findings of this study are available from the author upon reasonable request.
