# Supplementary material for: Efficacy of Cognitive Behavioral Therapy for Anxiety-Related Disorders: A Meta-Analysis of Recent Literature
Source: Curr Psychiatry Rep. 2022 Dec 19;25(1):19–30. doi: 10.1007/s11920-022-01402-8 (PMC9834105; doi:10.1007/s11920-022-01402-8)
Supplement: Supplementary file 1 — Supplementary file1 (DOCX 23 KB) [file 11920_2022_1402_MOESM1_ESM.docx]

| **Table 2- Effects of cognitive behavior therapy for adult anxiety-related disorders compared to placebo controlled trials** | | | | | | | | |
| --- | --- | --- | --- | --- | --- | --- | --- | --- |
|  |  |  |  |  |  |  |  |  |
| **Study** |  | **^N^comp** | **Effect size** | | | **Heterogeneity** | |  |
|  |  |  | ***g*** | **95% CI** | ***p^a^*** | ***I^2^*** | ***95% CI*** |  |
| Overall effects on anxiety-related disorders | | 10 | 0.24 | [0.06; 0.41] | 0.013* | 26% | [0.0%; 64%] |  |
| Effect size of CBT on PTSD |  | 7 | 0.14 | [ 0.02; 0.24] | 0.025* | 0% | [0.0%; 71%] |  |
| Effect size of CBT on Depression |  | 7 | 0.15 | [-0.11; 0.40] | 0.215 | 36% | [0.0%; 73%] |  |
| Effect size of CBT-PTSD on Depression | | 6 | 0.09 | [-0.12; 0.32] | 0.315 | 17% | [0.0%; 62%] |  |
| Follow-up |  | 7 | 0.09 | [-0.08; 0.28] | 0.249 | 0% | [0.0%; 71%] |  |
| Follow-up on PTSD |  | 6 | 0.08 | [-0.12; 0.29] | 0.339 | 0% | [0.0%; 74%] |  |
| Subgroup analyses |  |  |  |  |  |  |  | ***p^b^***  Between subgroups |
|  |  |  |  |  |  |  |  |  |
| Treatment Format |  |  |  |  |  |  |  |  |
|  | Individual | 4 | 0.20 | [ 0.01; 0.38] |  | 0% | [0.0%; 85%] | 0.74 |
|  | Group | 6 | 0.25 | [-0.09; 0.58] |  | 53% | [0.0%; 81%] |  |
|  |  |  |  |  |  |  |  |  |
| Mode of Assessment |  |  |  |  |  |  |  |  |
|  | Self report | 3 | 0.13 | [-0.21; 0.47] |  | 0% | 0.0%; 90%] | 0.27 |
|  | Clinician | 7 | 0.28 | [ 0.02; 0.53] |  | 38% | [0.0%; 74%] |  |
|  |  |  |  |  |  |  |  |  |
| Analysis type |  |  |  |  |  |  |  |  |
|  | Completers | 3 | 0.17 | [-0.75; 1.09] |  | 26% | [0.0%; 92%] | 0.72 |
|  | ITT | 7 | 0.25 | [ 0.04; 0.46] |  | 36% | [0.0%; 74%] |  |
|  |  |  |  |  |  |  |  |  |
| Comparison Condition |  |  |  |  |  |  |  |  |
|  | Other Psych. Placebo | 6 | 0.36 | [ 0.09; 0.62] |  | 27% | [0.0%; 70%] | *0.04 |
|  | PCT | 4 | 0.11 | [-0.11; 0.34] |  | 0% | [0.0%; 85%] |  |
|  |  |  |  |  |  |  |  |  |
| Comparison Condition for PTSD |  |  |  |  |  |  |  |  |
|  | Other Psych. Placebo | 3 | 0.18 | [ -0.10; 0.47] |  | 0% | [0.0%; 90%] | 0.45 |
|  | PCT | 4 | 0.11 | [- 0.11; 0.34] |  | 0% | [0.0%; 85%] |  |
|  |  |  |  |  |  |  |  |  |
|  |  |  |  |  |  |  |  |  |
| Participants in PTSD studies |  |  |  |  |  |  |  |  |
|  | Active-duty military participants | 4 | 0.14 | [-0.12; 0.39] |  | 0% | [0.0%; 85%] | 0.98 |
|  | Non-military participants | 3 | 0.13 | [- 0.13; 0.40] |  | 0% | [0.0%; 89%] |  |
|  |  |  |  |  |  |  |  |  |
| Meta regression |  |  |  |  |  |  |  |  |
|  |  |  | β | ***p*** |  |  |  |  |
|  | Number of therapy sessions as predictor of the effect size |  | -0,03 | 0.189 |  |  |  |  |

^a^ *The p value in this column indicate the effect size was significant*

^b^ *The p value in this column indicate* significant group differences
